# Perioperative adherence to continuous positive airway pressure and its effect on postoperative nocturnal hypoxemia in obstructive sleep apnea patients: a prospective cohort study

**DOI:** 10.1186/s12871-021-01371-0

**Published:** 2021-05-11

**Authors:** Colin Suen, Jean Wong, Kahiye Warsame, Yamini Subramani, Tony Panzarella, Rida Waseem, Dennis Auckley, Rabail Chaudhry, Sazzadul Islam, Frances Chung

**Affiliations:** 1 Department of Anesthesia and Pain Management, Toronto Western Hospital, University Health Network, University of Toronto, MCL 2-405, 399 Bathurst St., Toronto, ON M5T2S8 Canada; 2 Department of Anesthesia and Pain Management, Women’s College Hospital, University of Toronto, Toronto, ON Canada; 3 Dalla Lana School of Public Health, University of Toronto, Toronto, ON Canada; 4 Department of Anesthesia and Perioperative Medicine, London Health Science Centre, St. Joseph Health Care, Western University, London, ON Canada; 5 Division of Biostatistics, Dalla Lana School of Public Health, University of Toronto, Toronto, ON Canada; 6 Division of Pulmonary, Critical Care and Sleep Medicine, MetroHealth Medical Center, Case Western Reserve University, School of Medicine, Cleveland, OH USA

**Keywords:** Obstructive sleep apnea, Continuous positive airway pressure, Perioperative outcomes, Nocturnal hypoxemia, Adherence, Sleep disordered breathing

## Abstract

**Background:**

Although continuous positive airway pressure (CPAP) is the first line treatment for obstructive sleep apnea (OSA) patients, the perioperative adherence rate is unclear. The objective of this study was to determine the perioperative adherence rate of patients with OSA with a CPAP prescription and the effect of adherence on nocturnal oxygen saturation.

**Methods:**

This prospective cohort study included adult surgical patients with a diagnosis of OSA with CPAP prescription undergoing elective non-cardiac surgery. Patients were divided into CPAP adherent and non-adherent groups based on duration of usage (≥ 4 h/night). Overnight oximetry was performed preoperatively and on postoperative night 1 and 2 (N1, N2). The primary outcome was adherence rate and the secondary outcome was nocturnal oxygen saturation.

**Results:**

One hundred and thirty-two patients completed the study. CPAP adherence was 61% preoperatively, 58% on postoperative N1, and 59% on N2. Forty-nine percent were consistently CPAP adherent pre- and postoperatively. Using a linear fixed effects regression, oxygen desaturation index (ODI) was significantly improved by CPAP adherence (*p* = 0.0011). The interaction term CPAP x N1 was significant (*p* = 0.0015), suggesting that the effect of CPAP adherence varied on N1 vs preoperatively. There was no benefit of CPAP adherence on postoperative mean SpO_2_, minimum SpO_2_, and percentage of sleep duration with SpO_2_ < 90%. Use of supplemental oxygen therapy was much lower in the CPAP adherent group vs non-adherent group (9.8% vs 46.5%, *p* <  0.001).

**Conclusions:**

Among patients with a preoperative CPAP prescription, approximately 50% were consistently adherent. CPAP adherence was associated with improved preoperative ODI and the benefit was maintained on N1. These modest effects may be underestimated by a higher severity of OSA in the CPAP adherent group and a higher rate of oxygen supplementation in the non-adherent group.

**Trial registration:**

ClinicalTrials.Gov registry (NCT02796846).

**Supplementary Information:**

The online version contains supplementary material available at 10.1186/s12871-021-01371-0.

## Background

Obstructive sleep apnea (OSA) is a common sleep-related breathing disorder characterized by recurrent episodes of complete or partial upper airway obstruction during sleep. Among patients undergoing elective surgery, the prevalence of OSA is estimated to be at least 25% and as high as 80% in certain populations, such as patients undergoing bariatric surgery [1]. OSA has important perioperative implications as it is associated with an increased risk of cardiac and pulmonary complications, oxygen desaturations, difficult intubation, and in rare instances, death [2].

Many of the sequelae of OSA are strongly linked to the degree and duration of oxygen desaturation [3]. Preoperative indices of oxygen desaturation have been linked to postoperative complications. In one study, preoperative oxygen desaturation index (ODI) > 29 events/h, greater than 7% of sleep duration with SpO_2_ < 90%, and mean SpO_2_ < 93% were identified as thresholds predictive of increased postoperative complications [4]. Among patients with OSA, postoperative hypoxemia occurs mostly between postoperative nights two to five and may lead to serious consequences including poor wound healing, cardiac arrhythmias, and delirium [5, 6].

Continuous positive airway pressure (CPAP) therapy is currently the first line treatment for OSA. CPAP serves as a functional pneumatic upper airway splint, preventing airway collapse as well as the associated oxygen desaturation that may accompany respiratory events in sleep. With appropriate settings, CPAP is expected to treat sleep-disordered breathing and normalize oxygenation during sleep. The current guidelines from the American Society of Anesthesiologists (ASA) and Society of Anesthesia and Sleep Medicine (SASM) recommend continuing the use of CPAP at previously prescribed settings in the postoperative phase [7, 8]. At present, there is limited data to suggest that CPAP is protective in the postoperative setting for patients with OSA. Previously we demonstrated that perioperative auto-titrating positive airway pressure (APAP) therapy administered to newly diagnosed OSA patients (CPAP-naïve) significantly reduced postoperative apnea hypopnea index (AHI) and improved SpO_2_ in patients with moderate to severe OSA [9]. Despite continued use of CPAP perioperatively, patients may still experience postoperative hypoxic events [10]. Factors associated with the perioperative environment, including opioid use, fluid accumulation, and positional requirements may lead to previously prescribed CPAP settings becoming less effective [10]. To date, it is unclear whether preoperative settings for CPAP adherent patients are routinely sufficient to overcome postoperative physiological cardiorespiratory changes.

Similar to the low adherence with CPAP in the general population, [11] the adherence to CPAP therapy in surgical patients with newly diagnosed OSA is low - approximately 45% [9, 10]. There is a lack of knowledge regarding the perioperative adherence of CPAP in surgical patients with a pre-existing diagnosis of OSA and a CPAP prescription. The objective of this study was to investigate the rate of adherence to CPAP among surgical patients with a history of diagnosed OSA and a CPAP prescription. The study design was a prospective cohort study in order to replicate the real-world perioperative scenario. As well, we sought to specifically determine the effect of adherence to CPAP on postoperative outcomes such as postoperative ODI, mean SpO_2_, minimum SpO_2_, and percentage of sleep duration at SpO_2_ < 90% (CT90).

## Methods

### Ethics

Institutional Review Board (IRB) approval for this study (15-8946AE) was obtained through the University Health Network Research Ethics Board (University Health Network, 700 University Ave, Toronto, Ontario, Canada M5G1Z5) on June 12, 2015 by Dr. Alan Bartlett. All methods were performed in accordance with the relevant guidelines and regulations. Written informed consent was obtained from all subjects.

### Study design

This prospective cohort study was conducted at Toronto Western Hospital, University Health Network. The study was registered on ClinicalTrials. Gov registry (NCT02796846). Surgical patients with OSA and a CPAP prescription were followed to determine their adherence to CPAP. Nocturnal SpO_2_ was collected preoperatively, and on postoperative night 1 (NI), and 2 (N2).

### Study population

Participants were approached in the preoperative clinic and included based on the following criteria: 1) age over 18 yrs.; 2) a diagnosis of OSA with a prescription for CPAP; 3) scheduled for a non-cardiac surgery (general surgery, orthopedics, urology, plastic, and spinal surgery); and 4) expected minimum postoperative stay of at least one night. All patients were admitted on the same day of surgery. Patients were excluded based on the following criteria: 1) unable or unwilling to give informed consent; 2) on supplemental oxygen preoperatively (daytime or nocturnal); 3) pregnant; 4) undergoing tonsillectomy, septoplasty, uvuloplasty, pharyngoplasty, tracheostomy; or 5) prolonged (> 48 h) postoperative mechanical ventilation was anticipated. The diagnosis of OSA was determined based on previous laboratory-based polysomnogram (PSG), or home sleep apnea study, and/or a prescription of CPAP for OSA. Participants were divided into 2 groups for the study analysis: CPAP adherent and CPAP non-adherent. Due to an inability to routinely obtain CPAP downloads in the preoperative clinic, CPAP adherence was defined preoperatively by self-reported use of CPAP > 4 h per night. When available, postoperative CPAP adherence postoperatively was determined by download, but otherwise by self-report.

### Study procedures

Overnight SpO_2_ was monitored by a pulse oximeter wristwatch (PULSOX-300i, Konica Minolta Sensing, Inc., Osaka, Japan). The oximeter PULSOx-300i has 1 Hz of sampling frequency, 3 s of averaging time, and 0.1% SpO2 resolution. Oximetry monitoring was performed on OSA patients at home before surgery (preoperative night), on postoperative N1, and N2. Patients were instructed to bring their own CPAP machine. In order to reflect usual “real world” clinical practice and maximize generalizability, postoperative analgesia, fluid management, and supplemental oxygen were administered according to routine standard practice and at the discretion of the primary team caring for the patient. As is the standard of practice in the institution for those who were non-adherent with CPAP at home, anesthesiologists and surgeons were to advise these patients to use their CPAP perioperatively at the previously prescribed setting. If clinically indicated, the health care team could order CPAP both preoperatively and postoperatively or refer patients to Sleep Medicine for further management. Indications for postoperative PAP therapy included respiratory events such as bradypnea, desaturation, observed apnea or hypopneas, and pain-sedation mismatch. Postoperative adverse events were determined based on patient chart review and are defined in Supplemental Digital Content 1.

### Study outcome measures

The primary outcomes were: 1) postoperative CPAP adherence, defined as an average CPAP use ≥4 h per night, and 2) overnight oximetry measured at baseline (preoperative), and postoperative N1, and N2. For adherence outcomes, patients were asked to report the time of donning and doffing of the CPAP. These times were corroborated with nursing records. Adherence was calculated based on the duration of CPAP use. The overnight oximetry parameters were processed using the Profox software (Florida, USA). These included mean SpO_2_, minimum SpO_2_, oxygen desaturation index (ODI) defined as average hourly number of desaturation episodes with at least 4% desaturation lasting at least 10 s, and percentage of sleep duration at SpO_2_ < 90% (CT90). Oximetry data was processed between 00:00 and 6:00. Postoperative adverse events were determined using pre-specified definitions from patient chart review (Supplemental Digital Content 1).

### Sample size estimation

When adherence is defined as greater than or equal to 4 h of nightly CPAP use, 46 to 83% of the general population with OSA are reported to be non-adherent with treatment [12]. We assumed a perioperative non-adherence rate of 60%. Based on data from our previous study [9], we assumed that postoperative ODI in CPAP adherent patients would be similar to APAP treated patients (14 ± 18 events/h), and that the ODI in CPAP non-adherent patients would be similar to control patients (32 ± 25 events/h). In order to detect this magnitude of difference with statistical power of 0.9 and alpha value of 0.05, the estimated total sample size was 90 using a t-test. Based on a 10% withdrawal rate, and 70% rate of oximetry completion, the number of recruited patients would be 142.

### Statistical analysis

The data was entered into a Microsoft Access database (Redmond, WA). Data processing and analyses were conducted using Stata 14.2 (StataCorp LP, College Station, TX); and RStudio Version 1.1.463. Descriptive statistics were employed to succinctly describe baseline demographic characteristics between CPAP adherent and non-adherent patients. The dataset was assessed for missing, duplicate, and miscoded values. Two-tailed parametric and non-parametric tests were carried out to analyze the differences between CPAP adherent and non-adherent patients perioperatively. Normally distributed continuous data were presented as mean ± SD, and comparisons between groups were carried out by two-sample independent *t*-tests. Skewed continuous data were presented as median (interquartile range), and comparisons between groups were carried out by Mann-Whitney U test (Wilcoxon Rank Sum test). Categorical (nominal) data were expressed and summarized as frequencies and percentages. To determine the association between categorical data the chi-square test or Fisher’s exact test was used. A *p*-value less than 0.05 was considered statistically significant.

Our primary explanatory variable, CPAP adherence, is time-varying, and given our study was observational, we used a fixed effects regression model to test the relationship between CPAP adherence and oxygen saturation. Fixed effects ignore between subject variation and focus only on within-subject variation [13]. Oxygen supplementation and time (preop, postop night 1, and postop night 2) were treated as variables in the model and were also fixed effects. We considered CPAP adherence × time interaction terms as adherence varied by time. In using fixed effects regression, both measured and unmeasured stable effects over time are adjusted in the analysis.

## Results

### Study population and baseline demographics

**Fig. 1:**
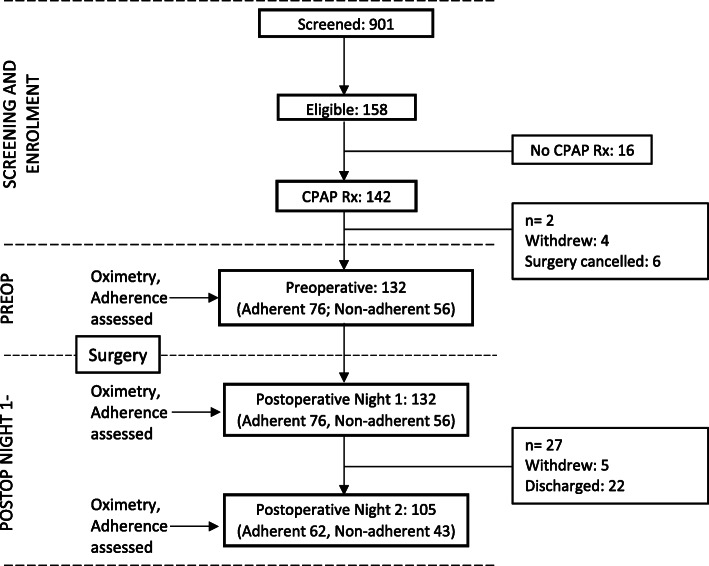
Study design: Patient recruitment and follow-up flow chart

The recruitment and follow-up of patients is shown in Fig. 1. A total of 901 patients were screened and consented, of which 158 patients were eligible for the study based on the inclusion criteria. Of those eligible, 89.9% of patients had a pre-existing CPAP prescription (Fig. 1). Among those with a CPAP prescription, ten patients dropped out of the study due to withdrawal from the study or surgery cancellation.

The baseline characteristics of CPAP adherent and non-adherent patients are presented in Table 1. Among the 132 total participants who completed the study, 88 (62.9%) were female with an average age of 51 ± 12 years, and a body mass index (BMI) of 44.7 ± 12.5 kg/m^2^. CPAP adherent patients had significantly higher BMI (47 ± 10 vs 41 ± 11 kg/m^2^, *p* <  0.001) and greater baseline AHI before CPAP therapy (41.1 (IQR 22.7, 77.0) vs 22.7 (IQR 14.2, 37.0) events/h, *p* = 0.002) than the CPAP non-adherent patients. There was a greater proportion of patients with severe OSA (AHI ≥ 30 events/h) in the CPAP-adherent vs non-adherent group (54.3 vs 25.0%, *p* = 0.010). (Table 1). There were no significant differences between CPAP adherent and non-adherent patients in ASA physical status, medical history, 48 h opioid consumption, types of surgery and types of anesthesia (Table 1).

**Table 1:** Baseline demographic data

Characteristics	Adherent (***N*** = 76)	Non-adherent (***N*** = 56)	***P*** Value
**Age (years)**	51 ± 11	51 ± 15	0.818
**Sex**			0.280
Male	23 (30.3%)	22 (39.3%)	
Female	53 (69.7%)	34 (60.7%)	
**BMI (kg/m**^**2**^**)**	47 ± 10	43 ± 11	0.017
**ASA Physical Status**			0.594
2	4 (5.3%)	6 (10.7%)	
3	68 (89.5%)	48 (85.7%)	
4	4 (5.3%)	2 (3.6%)	
**Medical History**			
Cardiovascular Disease^a^	40 (52.6%)	30 (53.6%)	0.915
Diabetes	24 (31.6%)	21 (37.5%)	0.478
GERD	34 (44.7%)	23 (41.1%)	0.674
Smoker	29 (38.2%)	15 (26.8%)	0.171
Asthma/COPD	19 (25.0%)	13 (23.2%)	0.813
Hypothyroidism	10 (13.2%)	8 (14.3%)	0.852
Arthritis	33 (43.4%)	21 (37.5%)	0.494
**AHI**^b^**, (events/h)**	36.1 (22.7, 74.0)	24.4 (16.2, 38.7)	0.031
**OSA Severity**			0.051
Mild	9 (14.5%)	11 (25.6%)	
Moderate	18 (29.0%)	18 (41.9%)	
Severe	35 (56.5%)	14 (32.6%)	
**Type of Surgery**			0.081
General	8 (10.5%)	6 (10.7%)	
Bariatric	54 (71.1%)	29 (51.8%)	
Orthopedic	5 (6.6%)	8 (14.3%)	
Spine	8 (10.5%)	11 (19.6%)	
Urology	1 (1.3%)	–	
Brain	–	2 (3.6%)	
**Type of Anesthesia**			0.441
General	59 (95.2%)	39 (90.7%)	
Spinal/Regional	3 (4.8%)	4 (9.3%)	
**48 h opioid consumption (mg)** ^c^	145.0 (90.0, 195.0)	105.6 (61.0, 186.3)	0.456
**Supplemental Oxygen Therapy**			
Postoperative Night 1	6/61 (9.8%)	20/43 (46.5%)	<.001
Postoperative Night 2	1/59 (1.7%)	2/42 (4.8%)	0.569

Data are represented as mean ± SD or median (IQR), or as otherwise indicated. OSA severity defined as mild (5 ≤ AHI < 15), moderate (15 ≤ AHI < 30), or severe (AHI ≥ 30) ^a^Cardiovascular disease includes hypertension, angina, myocardial infraction, heart failure, peripheral vascular disease, valvular disease, stroke, coronary revascularization, atrial fibrillation, ventricular tachycardia, supraventricular tachycardia, ventricular premature beats, atrioventricular block, and cardiomyopathy. ^b^Baseline AHI prior to CPAP therapy**.** ^b^Opioid consumption was reported as oral morphine equivalents (mg). *ASA* American Society of Anesthesiologists; *BMI* body mass index; *COPD* chronic obstructive pulmonary disease. *CPAP* continuous positivity airway pressure; *GERD* gastroesophageal reflux disease. *IQR* interquartile range. Two sample independent t-tests or Wilcoxon rank-sum test and chi-squared test, or Fisher’s exact tests were conducted to examine differences in baseline characteristics between adherent and non-adherent patients. Adherence is defined as an average CPAP use ≥4 h/night

### Perioperative CPAP adherence

**Fig. 2:**
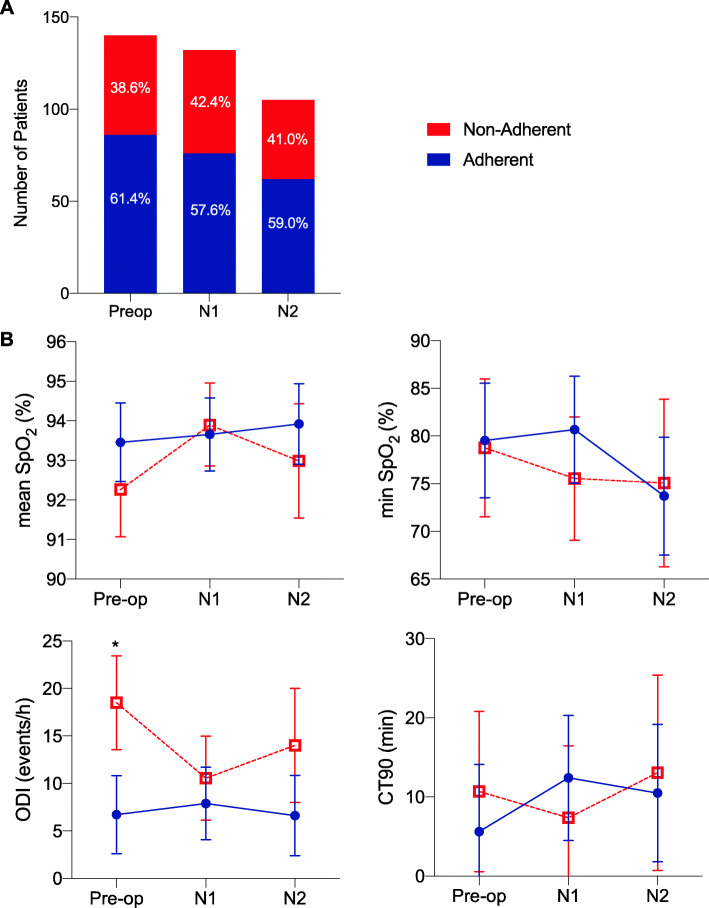
Perioperative CPAP adherence and corresponding nocturnal oxygen saturation in CPAP adherent versus non-adherent OSA patients. **a** Perioperative CPAP adherence patterns. **b** Oxygen saturation parameters measured by overnight oximetry. Preop = pre-operative night; *N* = postoperative night; Adherent = CPAP adherent; Non-adherent = CPAP non-adherent, ODI = oxygen desaturation index, CT90 = cumulative time percentage with SpO2 < 90%, Data represented as mean with 95% CI, * *p* <  0.05 vs adherent

Among patients with CPAP prescription, 61.4% were adherent preoperatively (Fig. 2a). In the postoperative period, on N1, 57.6% were adherent; and on N2, 59.0% were adherent.

Among 132 subjects who completed the study, 64 patients (48.5%) were consistently adherent to CPAP, defined as CPAP usage ≥ 4 h on all pre and postoperative nights (Supplemental Digital Content 2). Forty-seven patients (35.6%) were consistently non-adherent with CPAP usage < 4 h on all pre- and postoperative nights. Nineteen patients (14.4%) demonstrated partial non-adherence, which was defined as CPAP usage < 4 h on 1 or more nights. Fourteen patients (10.6%) who were preoperatively adherent demonstrated postoperative partial non-adherence. Six (4.5%) patients who were previously non-adherent preoperatively were placed on CPAP postoperatively on one or more nights due to oxygen desaturation by the health care team. Details of the longitudinal perioperative CPAP adherence are further illustrated in Supplemental Digital Content 2.

### Perioperative overnight Oximetry

Unadjusted, cross-sectional analysis comparing CPAP adherence vs non-adherence is presented in Supplemental Digital Content 3. Overall, CPAP adherence vs. non-adherence was associated with significantly higher preoperative minimum SpO_2_ (83 vs 79%, *p* = 0.001), lower ODI (4.3 vs 11.8 events/h, *p* <  0.001), and lower CT90 (0.5 vs 3.6%, *p* < 0.001) (Supplemental Digital Content 3).

Perioperative oxygen saturation parameters between CPAP adherent and non-adherent patients were analyzed using a linear regression fixed effects model, which only utilizes within-subject variation, and considers CPAP adherence as a time-varying covariate (Table 2). At the preoperative baseline, CPAP adherence versus non-adherence was associated with significantly lower ODI (6.71 [95% CI 2.60–10.83) vs 18.51 [95% CI 13.57–23.45] events/h, *p* = 0.0011), but no significant differences in mean SpO_2_ (93.5% vs 92.3%, *p* = 0.16), minimum SpO_2_ (79.5 vs. 78.8%, *P* = 0.88) or CT90 (5.61 vs 10.68%, *p* = 0.49) (Fig. 2b).

**Table 2:** Linear regression for perioperative overnight oximetry using a linear fixed effects model

Variable	Parameter estimate	SEM	T-statistic	***P***-value
**Mean SpO**_**2**_				
Intercept	92.98	1.6	58.17	< 0.0001
CPAP Adherence	0.54	0.78	0.69	0.49
N1 vs. Pre-op	1.13	0.495	2.27	0.025
N2 vs. Pre-op	1.12	0.56	3.01	0.076
O_2_ therapy	1.69	0.561	3.01	0.003
**Minimum SpO**_**2**_				
Intercept	84.45	9.6	8.8	< 0.0001
CPAP Adherence	2.15	4.7	0.46	0.65
N1 vs. Pre-op	−2.04	2.98	−0.69	0.49
N2 vs. Pre-op	−7.45	3.76	−1.98	0.0497
O_2_ therapy	−6.17	3.37	−1.83	0.070
**ODI**				
Intercept	18.75	6.63	2.83	0.0055
CPAP Adherence	−11.8	3.55	−3.33	0.0011
N1 vs. Pre-op	−7.96	2.32	−3.44	0.0008
N2 vs. Pre-op	−4.51	3.49	−1.29	0.19
CPAP adherence × N1	9.14	2.81	3.25	0.0015
CPAP adherence × N2	4.4	2.88	1.53	0.13
O_2_ therapy	−0.78	2.56	−0.3	0.76
**CT90**				
Intercept	2.95	13.53	0.22	0.83
CPAP Adherence	−0.35	6.62	−0.05	0.96
N1 vs. Pre-op	0.22	4.22	0.05	0.96
N2 vs. Pre-op	0.083	5.3	0.02	0.99
O_2_ therapy	−4.53	4.75	−0.95	0.34

Statistical analysis using linear fixed effects model where the time-varying covariate CPAP adherence only utilizes within-subject variation. By definition the time invariant covariates are assumed stable over time, and thus are removed from consideration. Abbreviations: *CPAP* continuous positive airway pressure, *ODI* oxygen desaturation index, *CT90* cumulative time percentage with SpO2 < 90%, *N1* postoperative night 1, *N2* postoperative night 2, *SEM* standard error of the mean.

Based on the linear fixed effects regression model, for the parameter ODI, we observed a statistically significant interaction between CPAP adherence and N1 (*p* = 0.0015) but not N2, which suggests differential effects of CPAP adherence in the postoperative period. For the parameters, mean SpO_2_, minimum SpO_2,_ and CT90, we performed a test of the interaction between compliance and night [i.e., main effects compliance, night, compliance × night interaction and O_2_ therapy) on 2 degrees of freedom]. There were no significant statistical interactions between CPAP adherence and time for mean SpO_2_, minimum SpO_2,_ or CT90. This led us to consider the simpler main effects model listed shown in Table 2. Adjusted mean values for ODI, mean SpO_2_, minimum SpO_2,_ and CT90 are shown in Supplemental Digital Content 4.

### Postoperative oxygen therapy

On N1, use of supplemental oxygen therapy was much lower in the CPAP adherent group vs non-adherent group (9.8% vs 46.5%, *p* < 0.001) (Table 1). On N2, no significant differences in supplemental oxygen therapy occurred between the two groups (1.7 vs 4.8%, *p* = 0.557) (Table 1). Supplemental O_2_ therapy on any postoperative night predicted an increase in mean SpO_2_ by 1.69 ± 0.56% (*p* = 0.003), but no significant differences for minimum SpO_2_, ODI, or CT90 (Table 2). Since oxygen therapy at baseline was an exclusion criterion, no patients received supplemental O_2_ therapy preoperatively.

### Postoperative complications

There were no significant differences in total postoperative adverse events among CPAP adherent vs non-adherent patients (38% vs 41%) (Supplemental Digital Content 5). No significant differences were observed in cardiovascular, respiratory, or gastrointestinal events. Interestingly, there was a significantly lower proportion of CPAP adherent patients vs non-adherent with inadequate pain control (11.1% vs 25.9%, *p* = 0.03) (Supplemental Digital Content 5).

## Discussion

This study describes the rate of perioperative CPAP adherence patterns among surgical patients with an established diagnosis of OSA and a pre-existing CPAP prescription. We observed a CPAP adherence rate of 61% preoperatively, 58% on N1, and 59% on N2. Only 49% of patients were consistently adherent to CPAP throughout the first 2 postoperative nights, while 4.5% of patients were non-adherent at baseline but received CPAP treatment postoperatively**.** Longitudinal analysis on ODI revealed a significant statistical interaction between CPAP adherence and N1, but not N2, suggesting differential effects of postoperative CPAP adherence. However, any benefit of CPAP adherence on N1 and N2 was not significant for mean SpO_2_, minimum SpO_2_, and CT90. Patients in the CPAP non-adherent group were three-fold more likely to receive supplemental O_2_ therapy.

Overall, the CPAP adherence rates reported were within the range of previously reported rates of 40–70% in the general population [14]. The present study demonstrates a small amount of variability of CPAP adherence in the postoperative period, where 10.6% who were preoperatively adherent demonstrated postoperative partial non-adherence. Conversely, only 4.5% of patients who were preoperatively non-adherent were placed on CPAP postoperatively on one or more nights and wore the device for at least 4 h. Several factors such as stress, anxiety, and postoperative discomfort resulting from pain, nausea, and vomiting, claustrophobia, dry mouth/nose, skin sores, aerophagia/bloating or perceived ineffectiveness likely contribute to postoperative nonadherence [9]. Some patients may be required to use hospital supplied CPAP equipment (failure to bring their own device), resulting in lack of heated humidification or ill-fitting CPAP interfaces, both are known to negatively impact CPAP adherence. Finally, preoperative adherence was based on the conventional longitudinal definition of > 4 h of use on > 70% of nights. However, the limited number of postoperative nights would not be adequate to determine adherence based on the definition above and therefore, the postoperative adherence rate is more sensitive to night-to-night variability.

Using longitudinal statistical analysis, a linear regression analysis showed a slight increase in ODI from preoperative baseline to N1 in the adherent group. In contrast, the opposite trend occurred for the non-adherent group, with ODI decreasing nearly 50% from baseline to N1. Previous reports have observed worsening of sleep apnea severity and hypoxemia in those with known OSA, despite the use of CPAP therapy postoperatively in some patients [10, 15]. Perioperative factors such as opioid administration, sedatives, and intravenous fluids may augment patient predisposition to sleep apnea by exacerbating upper airway collapse, depressing the arousal response, and intensifying rostral fluid shifts leading to upper airway edema and reduced patency [16]. These same factors could render preoperative CPAP settings, previously shown to be effective at controlling the patient’s OSA, less effective following surgery [10]. In contrast, auto-titrating CPAP devices has demonstrated efficacy in reducing postoperative nocturnal hypoxemia, possibly by adapting pressures to counter perioperative changes on upper airway collapsibility [9].

In order to replicate the real-world clinical scenario, supplemental O_2_ therapy was administered at the healthcare providers’ discretion. On N1, we found that the non-adherent group were three-fold more likely to receive supplemental O_2_ therapy than the adherent group. Additionally, we observed a reduction in postoperative versus baseline ODI in the non-adherent group to values comparable to adherent (10.56 vs 7.89, *p* = 0.46). Using linear regression modeling, supplemental O_2_ therapy accounted for a mean increase in SpO_2_ by approximately 2%. We have previously shown that postoperative O_2_ therapy improves AHI and oxygen saturation in patients with OSA [17]. It is plausible that CPAP non-adherent patients were more likely to desaturate or perceived to be at higher risk of desaturation in the postoperative period prompting their providers to prescribe O_2_ therapy. It is important to highlight that patient care providers did not have access to the overnight oximetry readings. The decision to administer supplemental O_2_ therapy was informed by clinical judgement and routine nursing spot checks for vitals. These factors could limit the ability to detect differences in the oximetry parameters between the CPAP adherent and non-adherent groups and contribute to the perceived normalization of oximetry parameters during hospitalization in the non-adherent group.

Another key caveat is that O_2_ therapy may cause CO_2_ retention and mask the detection of hypercapnia in some patients [18, 19]. Eleven percent of OSA patients were previously shown to have hypercapnia with postoperative O_2_ therapy [16]. The unintended consequence of O_2_ therapy is that a “normal” SpO_2_ could mask the timely recognition of hypoventilation which may spiral into hypercarbia, CO_2_ narcosis, respiratory failure, and even death [20]. Other forms of monitoring aside from oximetry such as continuous capnography could potentially aid in the early recognition of ventilatory abnormalities and may form the basis for future clinical studies [21].

In the present study, baseline AHI of all patients from their laboratory-based polysomnography were obtained before their CPAP prescription. Notably, the CPAP adherent group had severe OSA (mean AHI: 41.4 events/h) versus the non-adherent group with moderate OSA (AHI: 22.7 events/h). Thus, the non-adherent group represents a milder OSA phenotype that may be less prone to oxygen desaturation without CPAP treatment. A key finding is that using preoperative oximetry obtained within 1 week of surgery, ODI was 4.3 vs 11.8 events/h in the CPAP adherent vs the non-adherent group, respectively. Importantly, this suggests that the CPAP adherent patients generally had excellent response to treatment and had minimal fluctuation in ODI on N1 and N2, despite a higher pre-treatment severity of OSA. Several studies found an association between severe untreated OSA and higher postoperative respiratory and cardiovascular complications [22, 23]. It is plausible that CPAP adherence may be beneficial in preventing nocturnal oxyhemoglobin desaturation or postoperative complications in patients with severe OSA.

There are several limitations in this study. Ideally, a randomized controlled trial would best address the effect of CPAP adherence on nocturnal hypoxemia. However, it would be unethical to withhold treatment to OSA patients in whom CPAP is indicated. Thus, a prospective cohort study was the only feasible design to address the research question. Another limitation is that the study was designed to answer whether CPAP adherence improves nocturnal oxygen saturation based on the adherence definition of usage ≥4 h per night. It is unclear if this is adequate time on therapy to change postoperative oxygenation and other outcomes. Future studies should be designed to determine the appropriate time threshold of CPAP usage, utilizing objective data from machine downloads, for the perioperative period. This data may improve the accuracy of measuring adherence compared to self-reporting, which is prone to error and the Hawthorne effect. Second, sample size calculations assumed a larger effect size than what was measured in the study and there were dropouts on follow-up oximetry testing. These factors may have limited the statistical power to detect differences between CPAP adherence and the outcomes measured. Third, there was a high rate of oxygen supplementation in the CPAP non-adherent group which could potentially mitigate nocturnal hypoxemia in untreated OSA patients. Other limitations were in lack of data beyond N2 and duration of surgery. Future studies be designed to address these factors by monitoring adherence patterns and oximetry over prolonged postoperative inpatient stay. Also, the study was not powered to assess differences in postoperative complications between the CPAP adherent and non-adherent groups, definitive conclusions about CPAP use and postoperative complications should not be made.

## Conclusions

In conclusion, the preoperative CPAP adherence rate is approximately 60% in surgical patients with OSA and a CPAP prescription. Approximately 50% of OSA patients with a CPAP prescription were consistently adherent perioperatively. CPAP adherence was associated with improved preoperative ODI and the benefit was maintained on N1. These modest effects may be underestimated by a higher severity of OSA in the CPAP adherent group and a higher rate of oxygen supplementation in the non-adherent group.

## Supplementary Information

**Additional file 1.**

## Data Availability

The datasets generated during and analyzed during the current study are not publicly available due to the data containing information that could compromise research participant privacy/consent but are available from the corresponding author on reasonable request.
