# Supplementary material for: Perioperative adherence to continuous positive airway pressure and its effect on postoperative nocturnal hypoxemia in obstructive sleep apnea patients: a prospective cohort study
Source: BMC Anesthesiol. 2021 May 11;21:142. doi: 10.1186/s12871-021-01371-0 (PMC8111893; doi:10.1186/s12871-021-01371-0)
Supplement: Supplementary file 1 — Additional file 1. [file 12871_2021_1371_MOESM1_ESM.docx]

**Supplemental Materials**

**Supplemental Digital Content 1: Definition of postoperative adverse events**

**Supplemental Digital Content 2. Longitudinal CPAP adherence patterns by perioperative stage**

**Supplemental Digital Content 3. Unadjusted cross-sectional analysis of perioperative overnight oximetry in CPAP adherent and non-adherent patients**

**Supplemental Digital Content 4. Adjusted mean overnight oximetry values between CPAP adherent and non-adherence by perioperative stage**

**Supplemental Digital Content 5: Postoperative adverse events in CPAP adherent vs non-adherent**

**Supplemental Digital Content 1: Definition of postoperative adverse events**

| Adverse Event | Definition |
| --- | --- |
| Total postoperative adverse events | Number of patients with one or more postoperative adverse events |
| Respiratory adverse events | Any postoperative respiratory event |
| Serious postoperative adverse events | Adverse events which are potentially life threatening and requiring ICU monitoring |
| Severe hypoxemia | SaO_2_ <90% and/or cyanosis lasting more than 1 minute while patient was at rest and on room air, and requiring supplemental oxygen therapy |
| Hypercapnia | RR <8 /min and or PaCO_2_>50 mmHg and/or EtCO_2_>55 mmHg |
| Respiratory depression | Respiratory rate <8 /min and/or PaCO_2_>50 mmHg and/or EtCO_2_>55 mmHg. Required mask ventilation, and/or intubation and/or narcan reversal |
| Respiratory failure | Need for mechanical ventilation |
| Pulmonary edema | Difficulty breathing, coughing up frothy sputum and bilateral fluffy infiltrates on chest x-ray |
| Bronchospasm | Expiratory wheezing |
| Laryngospasm | Stridor with upper airway obstruction |
| Upper airway obstruction | Stridor with upper airway obstruction due to causes other than laryngospasm |
| Atelectasis | A local increase in density on chest X-ray, from thin platelike streaks to the collapse of an entire lung |
| Re-intubation | Patient has to be re-intubated after extubation in OR, PACU, ICU or ward |
| Myocardial infarction | The evolving changes in the ST-T segment and/or new Q wave on an electrocardiogram; symptoms of ischaemia plus abnormal serum levels of Troponin or symptoms of myocardial ischaemia plus new left bundle branch block |
| Cardiac arrest | The abrupt cessation of normal circulation of the blood due to failure of the heart to contract effectively during systole |
| Myocardial ischemia | New or more severe chest pain and >1 mm ST depression and/or inversion of the T wave on EKG |
| Congestive heart failure | New pulmonary edema on a chest radiograph or a diagnosis of congestive heart failure Dysrhythmia |
| Tachycardia | HR >120 beats/min for more than 15 min |
| Bradycardia | HR <50 beats/min for more than 15 min |
| Hypertension | Systolic blood pressure >200 mmHg for >5 min |
| Hypotension | Systolic blood pressure <80 mmHg for >5 min |
| Transient ischemic attack (TIA) | Abrupt onset of a focal neurological deficit lasting <24 h and resulting from cerebrovascular ischemia |
| Delirium | A disturbance of consciousness with impaired attention and disorganized thinking or perceptual disturbance that develops acutely. |
| Motor deficit | Unexpected inability to lift the upper or lower extremity for more than one hour (excluding spinal or epidural anesthesia) |
| Sensory deficit | Unexpected inability to feel pinprick in either the upper or lower extremity for more than one hour |
| Syncope | Partial or complete loss of consciousness with a spontaneous recovery |
| Somnolence | The state of drowsiness |
| Inadequate pain control | Persistent Pain with VAS 7 or higher; pain cannot be controlled by regular dose of narcotics; or further consultation with Pain Team is needed. |
| Prolonged oxygen therapy | The patients still requires oxygen therapy after discharge from PACU |
| Additional monitoring | Including oximetry, invasive arterial blood pressure monitoring, central venous pressure monitoring, and/or arterial blood gas monitoring |
| Additional need for treatment | Requiring prolonged oxygen therapy, addition monitoring and ICU transfer |
| Psychiatric adverse event | Including depression, anxiety and paranoia |
| GI adverse events | Including nausea and vomiting |
| Renal adverse events | Including renal failure, urinary retention, and urinary incontinence Other adverse events |

**Supplemental Digital Content 2. Longitudinal CPAP adherence patterns by perioperative stage**

| **Pre-op** | **Post-op Night 1** | **Post-op Night 2** | **Number** | **Percent** | **Trend over time** | **Overall pattern** |
| --- | --- | --- | --- | --- | --- | --- |
| Yes | Yes | Yes | 56 | 42.4 | Adherent | Consistently adherent |
| Yes | Yes | n/a | 8 | 6.1 | Adherent | Consistently adherent |
| No | No | No | 30 | 22.7 | Non-adherent | Consistently non-adherent |
| No | No | n/a | 17 | 12.9 | Non-adherent | Consistently non-adherent |
| Yes | Yes | No | 6 | 4.5 | Switched to non-adherence on N2 | Partially non-adherent |
| Yes | No | No | 5 | 3.8 | Switched to non-adherence on N1 | Partially non-adherent |
| Yes | No | Yes | 3 | 2.3 | Switched on N1 and N2 | Partially non-adherent |
| No | Yes | Yes | 3 | 2.3 | Switched to adherence N1 | Partially non-adherent |
| No | Yes | No | 2 | 1.5 | Switched on N1 and N2 | Partially non-adherent |
| Yes | No | n/a | 1 | 0.8 | Switched on N1 | Partially non-adherent |
| No | Yes | n/a | 1 | 0.8 | Switched on N1 | Partially non-adherent |
|  |  | **Total** | 132 | 100.0 |  |  |

Abbreviations: n/a = not available due to discharge from hospital. 64 (48.4 %) patients: consistently adherent; 47 (35.6%): consistently nonadherent; 21 (15.9%) partially non-adherent.

**Supplemental Digital Content 3. Unadjusted cross-sectional analysis of perioperative overnight oximetry in CPAP adherent and non-adherent patients**

|  | **Preoperative** | | | **Postoperative Night 1** | | | **Postoperative Night 2** | | |
| --- | --- | --- | --- | --- | --- | --- | --- | --- | --- |
|  | **Adherent** | **Non-adherent** | **P Value** | **Adherent** | **Non-adherent** | **P Value** | **Adherent** | **Non-adherent** | **P Value** |
| n | 75 | 51 |  | 61 | 40 |  | 50 | 24 |  |
| Mean SpO_2_ ^Ɨ^ | 93.8 ± 2.0 | 93.0 ± 2.2 | 0.039 | 93.1 ± 2.4 | 94.2 ± 2.3 | 0.041 | 93.1 ± 2.1 | 93.4 ± 2.2 | 0.606 |
| Minimum SpO_2_ ^Ɨ^ | 83.0 (78.0, 86.0) | 79.0 (71.0, 83.0) | 0.001 | 83.0 (79.0, 86.0) | 79.0 (71.5, 83.0) | 0.005 | 80.5 (71.0, 84.0) | 77.0 (74.5, 82.0) | 0.563 |
| ODI ^Ɨ^ | 4.3 (2.6, 9.9) | 11.8 (8.1, 20.5) | <0.001 | 5.0 (2.2, 11.7) | 8.0 (3.8, 12.5) | 0.246 | 5.8 (3.3, 10.3) | 6.4 (3.1, 15.0) | 0.366 |
| CT90^Ɨ^ | 0.5 (0.2, 4.4) | 3.6 (0.8, 12.7) | <0.001 | 3.3 (0.9, 10.4) | 2.0 (0.6, 7.3) | 0.329 | 2.9 (0.7, 9.2) | 4.5 (0.3, 8.0) | 0.867 |
| Supplemental O_2_^ǂ^ | -- | -- | -- | 6 (9.8%) | 20 (46.5%) | <0.001 | 1 (1.7%) | 2 (4.8%) | 0.569 |

CPAP, continuous positive airway pressure; CT90, percentage of total sleep duration with SpO2 <90%; ODI, oxygen desaturation index. Adherence is defined as an average CPAP use ≥ 4hrs per night at least 70% of nights. Independent sample t-test or Wilcoxon rank-sum (Mann-Whitney) were conducted to examine differences between CPAP adherent and non-adherent OSA surgical patients

^Ɨ^ Continuous variables were expressed as mean ± SD and median (IQR), ^ǂ^ Categorical variables were expressed as frequencies (%),

**Supplemental Digital Content 4. Adjusted mean overnight oximetry values between CPAP adherent and non-adherence by perioperative stage**

|  | **Adjusted mean** | |  |
| --- | --- | --- | --- |
|  | **Adherent** | **Non-adherent** | **p-value** |
| **Mean SpO2 (%)** |  |  |  |
| Pre-op | 93.46 (92.47, 94.45) | 92.26 (91.07, 93.45) | 0.16 |
| N1 | 93.66 (92.73, 94.58) | 93.89 (92.86, 94.96) | 0.78 |
| N2 | 93.92 (92.90, 94.94) | 92.99 (91.54, 94.43) | 0.32 |
| **Minimum SpO2 (%)** |  |  |  |
| Pre-op | 79.54 (73.53, 85.55) | 78.77 (71.55, 85.99) | 0.88 |
| N1 | 80.67 (75.06, 86.27) | 75.55 (69.08, 82.02) | 0.32 |
| N2 | 73.71 (67.53, 79.89) | 75.08 (66.29, 83.86) | 0.81 |
| **ODI (events/h)** |  |  |  |
| Pre-op | 6.71 (2.60, 10.83) | 18.51 (13.57, 23.45) | 0.0011 |
| N1 | 7.89 (4.07, 11.72) | 10.56 (6.13, 14.98) | 0.46 |
| N2 | 6.61 (2.38, 10.84) | 14.00 (7.99, 20.01) | 0.059 |
| **CT90 (min)** |  |  |  |
| Pre-op | 5.61 (-2.86, 14.08) | 10.68 (0.55, 20.82) | 0.49 |
| N1 | 12.40 (4.48, 20.31) | 7.37 (-1.71, 16.44) | 0.49 |
| N2 | 10.49 (1.81, 19.17) | 13.07 (0.71, 25.42) | 0.75 |

Regression analysis using a linear fixed effects model where the time-varying covariate CPAP adherence only utilizes within-subject variation. Means compared using F-test. Means adjusted for O2 supplementation, perioperative night and by adherence x time interaction. Values are represented as mean (95% CI)

**Supplemental Digital Content 5: Postoperative adverse events in CPAP adherent vs non-adherent patients**

| **Postoperative Complications** | **Adherent Preop, N=79** | **Non-adherent Preop, N=54** | **P Value** |
| --- | --- | --- | --- |
| ***Respiratory events*** | 7(8.9) | 4(7.4) | 1 |
| Desaturations | 6(7.6) | 4(7.4) | 1 |
| Upper Airway Obstruction | 1(1.3) | 1(1.9) | 1 |
| ***Cardiovascular events*** | 9(11.4) | 4(7.4) | 0.559 |
| Tachycardia | 2(2.5) | 0 | 0.514 |
| Ischemia | 1(1.3) | 1(1.9) | 1 |
| Hypotension | 3(3.8) | 0 | 0.646 |
| Hypertension | 4(5.1) | 2(3.7) | 1 |
| ***Neurological events*** | 13(16.4) | 16(29.6) | 0.071 |
| Confusion | 3(3.8) | 0 | 0.271 |
| Motor Deficit | 0 | 1(1.9) | 0.406 |
| Sensory Deficit | 0 | 1(1.9) | 0.406 |
| Sedation/Drowsiness | 4(5.1) | 1(1.9) | 0.648 |
| Inadequate pain control | 9(11.4) | 14(25.9) | 0.030* |
| ***Gastrointestinal events*** | 10(12.7) | 6(11.1) | 0.788 |
| Vomiting | 1(1.3) | 0 | 1 |
| Nausea | 10(13) | 6(11.1) | 0.788 |
| ***Total Complications*** | 30(38) | 22(41) | 0.748 |

Values are represented as total number of patients and proportion of adherent or non-adherent with complications in parentheses. * p < 0.05
